# Stress and adolescent well-being: the need for an interdisciplinary framework

**DOI:** 10.1093/heapro/daw038

**Published:** 2016-05-06

**Authors:** Inga Dora Sigfusdottir, Alfgeir Logi Kristjansson, Thorolfur Thorlindsson, John P Allegrante

**Affiliations:** 1 Department of Psychology, Reykjavik University, Reykjavik, Iceland; 2 Department of Health and Behavior Studies, Teachers College, Columbia University, New York, NY, USA; 3 Department of Social and Behavioral Sciences, School of Public Health, West Virginia University, Morgantown, WV, USA; 4 Faculty of Social and Human Sciences, University of Iceland, Reykjavik, Iceland; 5 Department of Sociomedical Sciences, Mailman School of Public Health, Columbia University, New York, NY, USA

**Keywords:** adolescence, bio-psychosocial model, harmful behavior, life course, strain, stress

## Abstract

Stress and strain among adolescents have been investigated and discussed largely within three separate disciplines: mental health, where the focus has been on the negative effects of stress on emotional health; criminology, where the emphasis has been on the effects of strain on delinquency; and biology, where the focus has been to understand the effects of stress on physiology. Recently, scholars have called for increased multilevel developmental analyses of the bio-psychosocial nature of risk and protection for behaviors of individuals. This paper draws on several different but converging theoretical perspectives in an attempt to provide an overview of research relevant to stress in adolescence and puts forth a new framework that aims to provide both a common language and consilience by which future research can analyze the effects of multiple biological, social and environmental factors experienced during specific developmental periods, and cumulatively over time, on harmful behavior during adolescence. We present a framework to examine the effects of stress on diverse behavioral outcomes among adolescents, including substance use, suicidal behavior, self-inflicted harm, and delinquency.

## INTRODUCTION

Adolescent substance use, self-harm, suicides and delinquency are large-scale problems in most economically advanced societies and of growing concern in developing countries. According to the most recent United Nations drug report, almost one-quarter of a billion people used illicit drugs in 2013 (United Nations Office on Drugs and Crime, 2015). Around 1 in 10 of these users will become addicted, but drug abuse kills around 200 000 people worldwide each year (United Nations Office on Drugs and Crime, 2015). In this context, it is important to note that most drug abusers initiate their use during adolescence (Sigfusdottir *et al.*, 2009). According to Selfharm UK, it is thought that as many as 13% of young people between the ages of 11 and 16 may intentionally try to hurt themselves at some point (Selfharm UK, 2015). New figures published in 2014 suggested a 70% increase in 10–14 year olds attending accident and emergency hospital departments for self-harm-related reasons over the preceding 2 years (Selfharm UK, 2015). In addition, both substance abuse and self-inflicted harm are positively related to delinquent behaviors such as engaging in stealing, vandalism and violence (Hirschi, 1969; Agnew, 2006). All these behavioral problems are also positively related to a host of additional issues in adolescents such as depressed mood (Mann *et al.*, 2014) and increased risk of school drop-out (Kristjansson *et al.*, 2008). Without attempting to mitigate the above-mentioned harmful behaviors, of an even graver concern are suicides, which have increased by 60% worldwide during the last four decades and are now among the three leading causes of death in this age group (Wasserman *et al*., 2005; www.Suicide.org).

Often, these problems arise when young people experience major stress in their lives and/or are the result of being born into adverse circumstances. Although quite a lot is known about the effects of stress, there are still major gaps in our knowledge, especially in relation to how stress affects physiological and emotional reactions, and harmful behavior. An important reason for this lack of understanding is the fact that studies of the social environment and human biology have developed largely as independent scientific disciplines. Currently, there is an emerging consensus that integrating factors at multiple biological and social levels is necessary in order to further our knowledge of human health and behavior (D'Onorfio and Lahey, 2010). It is, however, not an easy task to overcome the current disciplinary-based paradigms that are deeply rooted in the organizations of universities, funding agencies, and science policies. Often, studies that concern closely related topics, have developed along the lines of independent scientific disciplines in separate or even parallel ways, using different terminology for similar issues. An example of this can be found in public health and the lack of explicit theoretical and methodological linkages that exist between the disciplines of epidemiology and criminology in their work with marginalized populations (Akers and Lanier, 2009). The concepts with which the two disciplines work essentially have the same meaning but are addressed differently within the disciplines; more theoretically within criminology, and more practically within public health. Research on the important topic of stress is another good example of this. Various disciplines have identified stress as a key variable in relation to health and social problems. We do, however, lack research that brings together knowledge from the various scientific disciplines in a coherent study on stress. One reason for this is the divide between social sciences that focus predominantly on the social environment and behaviors and the natural sciences that concern the human body and biology. We simply do not have the kind of studies that include both refined measurement of social contexts and sophisticated measurement of biological processes that are relevant to understanding specific health problems. Only recently, in part because of the emergence of new technologies, have behavioral scientists begun to think simultaneously about the relevant social and biological mechanisms in the context of an integrated, multilevel developmental analytic framework in order to understand the processes and pathways through which the environment, social circumstances and biology *interact* to influence healthy adolescent development. As Cullen (Cullen, 2011) points out, social scientists can no longer pretend that biology is not a part of human behavior and thus an important part of harmful behavior. At the same time, we no longer need to fear that combining biology and social data will lead to ‘blaming the victim’, social engineering or biological reductionism. Ever since the human genome was sequenced in 2001, we have become more aware of the fact that the link between biology and environment is much more complex than we had thought. Studies have, for example, shown that increased maternal care given to rat pups permanently enhances the expression of a certain gene in areas of the brain that eventually affect the ways the animals react to stress (Francis, 1999; Weaver *et al.*, 2004). These studies have provided us with evidence that we have moved beyond the nature–nurture conundrum. We now know that just as our environment is potentially modifiable, our biology is flexible, and may be largely dependent on social processes, and that the two work in tandem to shape the individual and the life course (Rafter, 2008). In line with that, Francis and Kaufer (Francis and Kaufer, 2011) recently argued that we should finally abandon the nature versus nurture debate and focus on understanding the mechanisms through which our biology and environments are intertwined and affect each other throughout people's lives.

In this paper, we propose a multilevel developmental framework that we believe can be used to examine the influence of stress factors on diverse behavioral outcomes among adolescents, including substance use, suicidal behavior, self-inflicted harm and delinquency. Drawing on biological, social and health theories, as well as plethora of research on the effects of stress on physiology, emotions and behavior, we put forth a framework that brings together three different paradigms. We describe the effects of multiple environmental factors experienced during specific developmental periods, and cumulatively over time, on behavior during adolescence, and we elaborate on the bio-social link between stress, physiology, emotions and behavior. Our aim is not to put forth a holistic integrated theory on the effects of stress on adolescent behavior, but rather to call the attention of the scholarly communities to the overlap in knowledge, the increasingly critical importance of consilience and the potential benefit of making scholars in the various communities familiar with the works of each other.

## THREE PARADIGMS OF STRESS RESEARCH

Three main paradigms have been developed and used over several decades to examine and understand the effects of stress on health and behavior. The first one is rooted in biology and focuses on the concept of allostatic load (AL) to capture the cumulative biological burden exacted on the body from repeated attempts to adapt to life's demands (Seeman *et al.*, 2001). The second paradigm stems from the mental health literature and focuses on the negative effects of stress on emotional and mental health (Dohrenwend and Dohrenwend, 1969; Aneshensel *et al.*, 1991). The third paradigm is rooted in criminology/sociology of deviance and studies the effects of what is labeled ‘strain’ on delinquent behavior (Merton, 1938; Cohen, 1955; Cloward and Ohlin, 1960).

We argue that the main difference between these three paradigms on the effects of stress on health and behavior is that they study stress on different levels; physiological, psychological, behavioral and group or community levels using different theoretical perspectives, different terminology and often focusing on different variables. For example, the various disciplines have discussed the effects of stress and strain on separate outcomes. The health sciences have focused on the effects on physiological reactions, psychology on analyzing the effects on emotional reactions and mental health and some criminologists and sociologists on the effects of stress and strain on behavior, whereas other sociologists and criminologists have focused on the rates of sickness or crime within groups and the collective efficacy of communities or even populations to counteract health risks and problem behavior (Sampson *et al.*, 1999). The focus within different fields has also been on diverse levels at which stress/strain is created. Some sociologists and criminologists, for example, have focused on the larger structure of society and the links between social structure and the health-related problems of communities and populations, while research on stress and health within biology and mental health disciplines have placed greater emphasis on different stress exposures experienced by individuals, for example, in the form of negative life events or clinically diagnosed conditions such as depression or anxiety disorders. Hence, there are numerous social factors and conditions that may influence or increase the likelihood of stress and strain; originating within the broader organization of society, in negative life events that include both chronic stressors, such as persistent family conflicts, and acute events, such as a sudden death of a parent. We believe that considerably more knowledge on the processes of how stress leads to physiological, emotional and behavioral outcomes is a necessary prerequisite for understanding stress-related health problems. Below we discuss the different aspects of the three paradigms.

### Stress and physiology

The first paradigm is rooted in biology and focuses on the effects of stress on physiological reactions. While humans have evolved to cope well with the occasional instances of acute stress caused by life-threatening events, recurrent activation of the body's stress response, particularly if the individual lacks coping resources, may have harmful effects on the body's physiology in the long run. The hypothalamus–pituitary–adrenal axis (HPA) and the sympathetic adrenal medullary system (SAM) are considered primary mediators through which all stressors activate a common set of physiological pathways. These systems stimulate adaptation or ‘allostasis’ as defined by Sterling and Eyer (Sterling and Eyer, 1988). In the short term, allostasis allows us to adapt to a wide range of stressors, but when frequent or excessive demands push allostatic processes beyond their normal operating ranges, wear and tear at the cellular level follows. AL is the result, representing the cumulative impact of stressors on the body's regulatory systems, with AL contributing to outcomes such as poor mental and physical health outcomes (Danese and McEwen, 2012).

Prior research has empirically supported some of the pathways by which stress influences physiology. For example, low socioeconomic status and poor interpersonal relationship histories have been associated with increased AL in a number of community-based cohorts (Johnson *et al.*, 1992; Karlamangla *et al.*, 2002; Seplaki *et al.*, 2006) and prospective research has associated AL at baseline with increased risk for all-cause mortality, cardiovascular disease and declines in cognitive and physical functioning. Family environments, social conditions and psychological processes have in this way been shown to affect biological processes and biological functioning and predispositions influence the ways in which an individual selects and is shaped by the environment (Rutter, 2002; Repetti *et al.*, 2011). All-in-all, research to date suggests that there are multiple interconnected biological systems that respond to psychosocial stress and influence each other. Hence, minor alterations in one system due to psychosocial stress may influence the functioning of the other(s).

### Stress and emotions

Based on findings showing that stressful life events contribute to the onset and course of mental symptoms and disorders, the social stress model has guided efforts to examine social experiences and circumstances that are associated with variations in risk for mental health problems (Dohrenwend and Dohrenwend, 1969; Turner *et al.*, 1995; Pearlin, 1999). The underlying assumption of the model is that variations in stress exposure are closely related to individual life conditions and social circumstances. In line with this reasoning, Aneshensel (Aneshensel, 1992) called for a reorientation away from viewing stress as an isolated risk factor and toward its consideration as a link in a causal chain beginning with social conditions and ending with differences in risk for psychological distress. Numerous studies have supported the social stress model. Hence, both negative life events and chronic strain in the form of poverty, family conflict or abuse have been found to predict emotional problems (Dohrenwend, 1990; Aneshensel *et al.*, 1991; Aneshensel, 1992; Turner and Lloyd, 1999; Ross, 2000).

The process through which stress affects emotions is obviously complicated. Understanding the underlying biological systems is a vital piece in this puzzle. A body of research has implicated disturbances in the HPA axis stress response system in the development of depression (Murray *et al.*, 2010). Researchers have also suggested that perturbations of cortisol may represent a risk factor for the development of depression. Thus, Goodyer *et al*. (Goodyer *et al.*, 2000) studied a sample of high-risk adolescents and found that the occurrence of one or more very high morning cortisol values over several days of salivary collection predicted the onset of depressive disorder in the subsequent 12 months. Adam *et al*. (Adam *et al.*, 2010) reported similar findings, showing that a larger increase in cortisol in the 30 min after waking (i.e. the cortisol awakening response) predicted onsets of depression over the subsequent year among adolescents at risk for depression due to high levels of neuroticism. High levels of negative mood and life events at baseline also independently predicted onsets of depression in the same study.

### Stress, social environment and social structure

It is now widely recognized that the social environment and social relationships can have powerful effects on health and behavioral outcomes. However, several studies underscore the complexity of capturing the social influences at various levels (Brooks-Gunn *et al.*, 1993; Duncan and Raudenbush, 1999; Sampson, *et al.*, 1999, 2002; Bernburg *et al.*, 2009a, b, c). Colvin *et al*. (Colvin *et al.*, 2002) emphasize that it is important to note that coercion can happen both at the micro level of interpersonal relations and at the macro level, where it includes economic and social pressure, created by social circumstances such as structural poverty, unemployment and conflict among groups. Thus, strain and conflict at the social and the neighborhood levels may influence the level of stress among groups or individuals. Merton's influential strain theory is good example of this complexity of levels. His (Merton, 1938) classic theory of anomie describes how shared ideology of equal opportunity conflicts with cultural and social constraints that reduce or even hinder certain groups to achieve desirable social goals and may cause strain that is experienced as stress at both the group and individual level. Thus, Merton's theory of anomie and strain has inspired several sociological and criminological theories of the influence of social conditions on individuals (Merton, 1938; Cohen, 1955; Cloward and Ohlin, 1960). Merton's (Merton, 1938) theory of anomie also suggests that these cultural and social constraints may cause conflict and strain by hindering certain groups of adolescents to achieve desirable social goals. Researchers that have focused on these group effects of Merton's theory have described how community characteristics influence the life of children and adolescents over and beyond their individual-level experiences (Brooks-Gunn *et al.*, 1991, 1993; Kawachi *et al.*, 1997; Sampson *et al.*, 1999; Leventhal and Brooks-Gunn, 2000; Bernburg *et al.*, 2009a).

Below, we discuss the individual and the community aspects of the social paradigm separately. But before we do that, we would like to make two points regarding this issue. First, while the physiological and psychological paradigms focus on the individual as a unit of analysis, the sociological paradigm operates on two different levels (Duncan and Raudenbush, 1999; Sampson *et al.*, 1999; Billari, 2015). Recent advances in multilevel modeling have made it possible to analyze both the group and the individual level. In other words, we now can analyze the group level controlling for individual-level effects. Secondly, the same social mechanisms can operate on two different levels, for example, family conflict may exist between individual spouses, but they may also be influenced by community processes (Bernburg *et al.*, 2009c).

#### Individual-level effects

Several individual-level theories of stress research, which build on Merton's anomie theory, focus on the effects of strain on delinquent behavior. In 1992, Agnew put forth a revised version of strain theory, which he calls general strain theory (GST). GST combines aspects from different previously developed theories, including those on stress, equity/justice and aggression, to explain the effects of strain on harmful behavior among adolescents. Unlike prior strain theories, GST argues that adolescents are not only concerned with future goals of monetary success and middle class status but are also concerned about more immediate goals, such as doing well at school and being popular among peers. The theory proposes that social conditions may cause strain or blockage that frustrates adolescents and may lead to harmful behaviors.

While Agnew recognizes that there are many opportunities for individuals to experience strain, GST subsumes strain under three broad categories. First, strain may arise because individuals fail to achieve goals that they value. Secondly, GST maintains that strain arises if individuals experience threat or actual removal of valued stimuli, i.e. when individuals lose something that they value, for example, a boy- or girlfriend or when they need to leave their school and attend a new one. Thirdly, strain may emanate from the presentation of negative situations or events. This type of strain reflects the problems that arise for individuals when they experience adverse situations that they cannot legally escape from, such as family conflict, victimization or child abuse. GST proposes that adolescents are sometimes pressed into delinquency by negative emotional reactions that result from strain. In response to strain and its consequent negative emotional states, therefore, adolescents can respond with acts of theft, violence, vandalism and drug and/or alcohol use. Hence, GST can potentially explain a diverse range of delinquent behaviors, and provides an appropriate framework in the discussion on the effects of stress on multiple different outcomes, such as substance use, self-harm, suicides and delinquency.

A number of studies have provided support for GST, showing that strain leads to harmful behavior through negative emotional reactions. For the first 10 years, tests of the theory mainly focused on anger as the critical emotional reaction (Mazerolle and Piquero, 1998; Aseltine *et al.*, 2000; Mazerolle *et al.*, 2000; Capowich *et al.*, 2001).

This was because anger results when individuals blame their adversity on others. It increases the individual's level of perceived injury, creates a desire for retaliation, energizes the individual for action and lowers inhibitions. However, results on the effects of strain on delinquent behavior mediated through anger were somewhat mixed. While extensive evidence has shown that higher levels of anger in adolescence are associated with a host of adverse psychosocial outcomes during that time period and in later life (e.g. Evans and English, 2002; Wittmann *et al.*, 2008; Midei and Matthews, 2009; Sigfusdottir and Silver, 2009; Sigfusdottir *et al.*, 2010), other studies have not revealed anger to be a key mediator (Unnever *et al.*, 2004).

In line with these findings, scholars have pointed out that anger is not the only emotion likely to arise under stress and highlighted the importance of furthering our understanding of *different emotions* as mediating factors in this relationship (Sigfusdottir *et al.*, 2004). In recent years, studies have shown that although anger and depressed mood are highly correlated emotions, comorbidity does not mean that these emotions are similar in their relations to behavioral outcomes (Sigfusdottir *et al.*, 2004, 2008; Asgeirsdottir *et al.*, 2011). Whereas anger energizes the individual for action, lowers inhibitions and hence increases externalizing behavior, depressed mood is not related to this type of behavior. Similarly, depressed mood is highly associated with certain kinds of (internalizing) behavior, such as suicidal ideation and self-harm, while anger is a much weaker predictor of those behaviors. These findings have added to our understanding of the implications of the interrelatedness between these phenomena, showing that whereas depressed mood and anger are overlapping phenomena, they are separate in their relations to behavioral outcomes. At the same time, they have revealed how complicated this process is, and the fact that the way adverse circumstances translate into behavior is still little understood. For example, a recent paper on family conflict/violence and sexual abuse, and suicidal ideation and attempt showed that even though depressed mood and anger were highly comorbid, co-occurring to a high degree, they differed in their behavioral outcomes; depressed mood was more strongly associated with suicidal ideation, whereas anger was more strongly related to suicidal attempts (Sigfusdottir *et al.*, 2013). Furthermore, the complexity of the associations between stress, emotional reactions and harmful behavior become apparent when considering the findings that; when controlling for anger, depressed mood is strongly related to suicidal ideation and remains also quite strongly related to suicidal attempt, but when controlling for depressed mood, anger is only related to suicidal attempt.

#### Community-level effects

Merton's (Merton, 1938) theory of Anomie suggests that some societal or neighborhood factors are contextual in the sense that they cannot be reduced to individual-level experience. They describe how community characteristics influence the life of children and adolescents over and beyond their individual-level experiences. It is therefore important to incorporate higher-level measures on the local community level and policy environment (e.g. counties, districts) that may now be studied in conjunction with individual-level outcomes using multilevel analysis techniques (e.g. hierarchical linear models, growth curve models, multilevel structural equation models). Such approaches provide an important way to study how community characteristics influence the life of children and adolescents in combination with individual characteristics. Studies on community-level stress have focused on important structural factors, such as community poverty, neighborhood instability, inequality and relative deprivation (Brooks-Gunn *et al.*, 1991, 1993; Kawachi *et al.*, 1997; Sampson *et al.*, 1999; Leventhal and Brooks-Gunn, 2000; Bernburg *et al.*, 2009a). For example, community levels of family conflict influence not only the likelihood of harmful behavior among adolescents that experience disruption personally, but also that higher aggregated community levels of disrupted family processes increase the likelihood of harmful behavior among all adolescents in such communities (Bernburg *et al.*, 2009c). This point is important, especially given the central role that families play in many theories of child and adolescent harmful behavior (Hirschi, 1969; Agnew, 1992; Sampson and Laub, 1993). The recent multilevel findings indicate that research on the effect of stress, including family conflict on adolescent behavior, should not be limited to individual-level analysis. Time and age also appear to matter in this respect; Odgers *et al*. have recently shown that neighborhood effects on child outcomes may be detected as early as from the age of 5, and that this association tends to increase over time (Odgers *et al.*, 2012). In order to fully understand the processes through which neighborhoods affect individuals, we need to focus not only on the link between neighborhood characteristics and behavioral outcomes, but also on the mediating mechanisms in the form of emotional and biological responses. Recently, Wallace (Wallace, 2012) made an important point by suggesting that disorder needs to invoke feelings of fear in order to affect peoples' health. Hence, it is necessary to study not only the main effects of neighborhood characteristics on outcomes, but to include possible mediating mechanisms, in the form of emotional reactions and biological responses. The framework we are putting forth proposes that stress, including neighborhood stress, affects behavior through both physiological and emotional reactions. Hence, the framework argues for the need to capture neighborhood characteristics all at once, is fit for neighborhood modeling and includes a variety of individual-level survey and biomarker measures. Recent findings indicate that research on the effect of stress, including family conflict on adolescent behavior, should not be limited to individual-level analysis, therefore adding a new dimension to previous research paradigms. In short, prior findings from multilevel analysis show that limiting research to the individual-level approach provides an incomplete account of the effects that the social environment has on children and adolescents.

## COMPLEX PATHWAYS

**Fig. 1:**
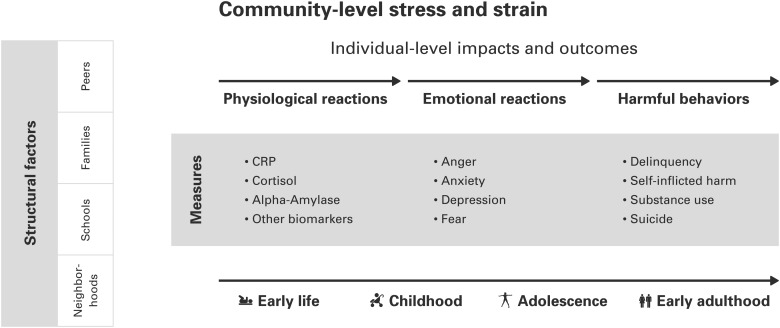
Community-level stress and strain and the proposed pathways to harmful behavior, with associated measures.

Each of the three paradigms above have guided studies showing that stress affects our physiology, emotions and behavior. However, the relationships between stress, physiology, emotions, behavior, and social structure are complex. Exactly how stress translates into outcomes such as harmful behavior is little understood. Hence, it is highly likely that whether or not an environmental stress becomes relevant to an individual does not only depend on how often a stressor occurs and how severe it is, but how strongly the individual physiologically, and emotionally, reacts to stress. To corroborate the view of how complicated this process is, recent studies on disruption of the HPA axis and cortisol production paint an inconsistent picture on its relations with emotional reactions (Tyrka *et al.*, 2010) and harmful behavior (Sondeijker *et al.*, 2007; Ruttle *et al.*, 2011). In order to come closer than previous work has in estimating how environmental stress and strain may affect biological responses among adolescents, the framework presented in Figure 1 proposes that stress and strain at the community and individual levels affect physiological and emotional reactions along the early life to childhood continuum that can result in harmful behavior during adolescence.

This proposed multilevel developmental framework argues for the importance of examining the impact of stress on biological systems and specific emotions, as well as the interplay between these factors in order to elucidate the relevant pathways to different behavioral outcomes. The framework suggests that future studies examine in detail the influence of cumulative as well as specific stressors on emotional reactions, while identifying essential moderators and the potential mediating role of the biological systems in this relationship and the development of these pathways across important developmental transitions. The arrows connecting these factors to harmful behavioral outcomes in adolescence represent the pathways of primary interest. Thus, stress experienced early in life may have implications for harmful behavior in adolescence, through physiological and emotional reactions.

## DISCUSSION

During the last 20 years, research with children and adolescents has linked stress to poor health and harmful behavior. Research has demonstrated that social conflict at various levels, ranging from societal levels to specific social groups, such as the family or peer groups, can increase stress that has harmful consequences for health and the well-being of children and adolescents. First, research at the societal level has shown how social environments can influence the consequences of stress. Secondly, research at the psychological level has demonstrated how stressful situations and life events interact with personal characteristics to produce harmful stress-related outcomes. Thirdly, research on stress at the biological level casts light on the biological and physiological mechanisms involved in the harmful influences of stress on human health. The diversity of concepts applied in the study of stress and strain mirrors the fact that the domain has been largely investigated and discussed within separate academic disciplines, each focusing on a certain level of analysis; with the biological sciences focusing on the effects of stress on physiological reactions, psychological sciences examining the effects of stress on emotional reactions and social sciences focusing on the effects of stress/strain on behavior. Within different fields, the focus has also been on diverse levels at which stress/strain is created, with sociologists and criminologists, for example, focusing on the larger structure of society and the links between social structure and population strain; they have mostly worked with the term strain and its effects on delinquency. Research on stress within health disciplines, however, has placed greater emphasis on different stress exposures experienced by individuals and their effects on individual mental and physical health.

There is no doubt that research at these different levels has furthered our understanding of the harmful effects of stress on the welfare of children and adolescents. But despite several decades of robust findings on the effects of adverse experiences on health and harmful behavior, major gaps still remain in our knowledge about the mechanisms through which adverse experiences work to increase the likelihood of poor health and harmful behavior during adolescence (Cullen, 1994; Colvin *et al.*, 2002). We know, for instance, that exposure to, and experience of, stress increases the odds for later negative development, including emotional problems, deficits in physical health and even harmful behaviors. What we do not know is whether these odds are cumulative, can be quantified or can be reversed with external supportive interventions during early developmental stages and later adolescence.

We believe that bringing together knowledge from the various scientific disciplines in a coherent study on stress is critical for advancing our understanding of threats to adolescent well-being. Such an approach would have important implications for policies within education, criminal justice and physical and mental health. We propose that in order to come closer than previous work has in estimating how environmental stress may affect biological responses that in turn lead to different emotions and behavior among adolescents, a comprehensive research approach must form the basis for future empirical studies. Moreover, it is important to design longitudinal studies where data on participants are collected at several times over the lifespan, including data drawn on key variables from the pre-birth period, such as maternal and intrauterine factors. Developing a research design that allows us to combine developmental approaches and various contexts in a comprehensive and effective way is one of the major challenges for future research (see Duncan and Raudenbush, 1999). Also, to provide a basis for developing a novel and comprehensive understanding of adolescent health and harmful behavior, it is important to examine both mediating and moderating effects of social–environmental predictors on physiological, emotional and behavioral outcomes. By taking such a theoretical approach, we would overcome the methodological weaknesses that many studies focusing on the early determinants of children's environment on later outcomes have faced. Moreover, as pointed out in a recent review, clinical samples do not provide a comprehensive understanding of confounding and comorbid factors, as the thresholds of discrete conditions are already defined, while cross-sectional studies cannot enhance our understanding of developmental processes (Thompson *et al.*, 2010). Thus, in addition to issues pertaining to research design, we argue that it is important to combine different theoretical paradigms of research on the effects of stress on behavioral outcomes.

It is important to capture variables from biological, individual and community levels in one comprehensive analysis. Such a holistic conceptual approach would allow us to disentangle how multiple environmental factors intertwine to produce greater odds for unhealthy development. To accomplish this task, we need to move beyond a narrow discipline-based approach by adding together viewpoints and methodological approaches from different fields. To facilitate such work, we need a universal language and uniform concepts and measures to describe similar processes that heretofore have been discussed using different terms within diverse disciplines. A prerequisite for such common language is a multilevel developmental framework, accompanied by empirical tests, telling us whether similar processes may be at work in different fields. The framework we have proposed provides such a prerequisite for investigating the effects of multiple-level factors, within the larger structure of society as well as in closer social circumstances of the individual, experienced during specific developmental periods, and cumulatively over time, on physiology, emotions and behavior in adolescence. Such a model would provide a unique opportunity to begin to understand the questions about whether the effects of stress may be conditioned by outside factors and what intervention approaches prove most beneficial in hindering harmful emotional and behavioral reactions to stress during adolescence.

## FUNDING

This work was supported by a Project Grant (206580-21-22-23) from RANNIS, the Icelandic Centre for Research and a Research Consolidator Grant (ERC-CoG-2014—No. 647860) from the European Research Council.
